# The potential of real-time analytics to improve care for mechanically ventilated patients in the intensive care unit: an early economic evaluation

**DOI:** 10.1186/s12962-020-00254-4

**Published:** 2020-12-11

**Authors:** Lytske Bakker, Katerina Vaporidi, Jos Aarts, William Redekop

**Affiliations:** 1 Erasmus School of Health Policy & Management (ESHPM), Erasmus University, Burgemeester Oudlaan 50, P.O. Box 1738, 3062 PA Rotterdam, The Netherlands; 2 Institute for Medical Technology Assessment (iMTA), Erasmus University, Burgemeester Oudlaan 50, 3062 PA Rotterdam, The Netherlands; 3 Department of Intensive Care, School of Medicine, University Hospital of Heraklion, University of Crete, Heraklion, Greece

**Keywords:** Cost- effectiveness analysis, Ventilation, Interactive ventilator support, Big data analytics, Intensive care unit

## Abstract

**Background:**

Mechanical ventilation services are an important driver of the high costs of intensive care. An optimal interaction between a patient and a ventilator is therefore paramount. Suboptimal interaction is present when patients repeatedly demand, but do not receive, breathing support from a mechanical ventilator (> 30 times in 3 min), also known as an ineffective effort event (IEEV). IEEVs are associated with increased hospital mortality prolonged intensive care stay, and prolonged time on ventilation and thus development of real-time analytics that identify IEEVs is essential. To assist decision-making about further development we estimate the potential cost-effectiveness of real-time analytics that identify ineffective effort events.

**Methods:**

We developed a cost-effectiveness model combining a decision tree and Markov model for long-term outcomes with data on current care from a Greek hospital and literature. A lifetime horizon and a healthcare payer perspective were used. Uncertainty about the results was assessed using sensitivity and scenario analyses to examine the impact of varying parameters like the intensive care costs per day and the effectiveness of treatment of IEEVs.

**Results:**

Use of the analytics could lead to reduced mortality (3% absolute reduction), increased quality adjusted life years (0.21 per patient) and cost-savings (€264 per patient) compared to current care. Moreover, cost-savings for hospitals and health improvements can be incurred even if the treatment’s effectiveness is reduced from 30 to 10%. The estimated savings increase to €1,155 per patient in countries where costs of an intensive care day are high (e.g. the Netherlands). There is considerable headroom for development and the analytics generate savings when the price of the analytics per bed per year is below €7,307. Furthermore, even when the treatment’s effectiveness is 10%, the probability that the analytics are cost-effective exceeds 90%.

**Conclusions:**

Implementing real-time analytics to identify ineffective effort events can lead to health and financial benefits. Therefore, it will be worthwhile to continue assessment of the effectiveness of the analytics in clinical practice and validate our findings. Eventually, their adoption in settings where costs of an intensive care day are high and ineffective efforts are frequent could yield a high return on investment.

## Background

Annual intensive care costs in the United States represent more than 13% of all hospital costs [1]. The costs of an intensive care unit (ICU) day per patient are high (i.e. €5,695) and an important factor that contributes to these high daily costs is whether or not patients receive mechanical ventilation [2]. Therefore, better management of mechanically ventilated patients could be a worthwhile investment when it reduces length of stay and their time on ventilation support.

One way to achieve better outcomes in the intensive care is by using analytics to process the huge amounts of monitoring data that are continuously collected in order to improve clinical decision-making [3]. Ventilation monitors in the ICU generate a wealth of data on a patient’s status and patient-monitor interaction. Ideally, this data can be used to help clinicians intervene promptly when the interaction between the patient and the monitor is poor. One example of poor interaction is when a patient tries but does not receive a breath. These so-called ‘ineffective efforts’ are reflected in the airway pressure and airflow data from the monitor [4]. When many ineffective efforts occur in a short period of time (> 30 ineffective efforts in 3 min.) it is referred to as an ineffective effort event (IEEV) which have been associated with higher hospital mortality, an increase in ICU length of stay of almost 10 days and prolonged time on mechanical ventilation [5]. Timely identification of ineffective effort events is crucial and early-warning systems using big data analytics have been portrayed as an important means to improve care for mechanically ventilated patients [4, 6] since the complexity and velocity required to process this data in real-time are beyond the capacities of humans such as healthcare professionals.

Real-time analytics of ventilation data would enable clinicians to identify IEEVs and intervene accordingly thereby shortening their duration and potentially reducing mortality risks and healthcare costs. Several types of interventions are recommended to improve the interaction between a patient and a mechanical ventilator such as, adjustment of ventilator settings, reducing sedation when managing pain and anxiety [7, 8] and adjustments in the management of bronchodilation [8]. Developing real-time analytics that identify IEEVs would enable clinicians to adopt these interventions currently already recommended when other forms of suboptimal interaction are present, identified manually for instance through waveform graphics [9]. However, large investments will need to be made in further research and development before these analytics could be implemented in clinical practice; the need for these investments can pose a major barrier for their development and future success. We aim to assist future development and clinical trial plans by identifying the performance requirements of the technology such as maximum costs or minimum efficacy. We performed a cost-effectiveness analysis in which we estimated how analytics that identify IEEVs in real-time could generate health improvements and/or financial savings.

## Methods

We used a decision tree model to assess the potential cost-effectiveness of analytics to detect IEEVs. Short term effects were estimated, such as hospital mortality and length of stay, but also long-term outcomes such as life years gained and quality adjusted life years gained (QALYs). Where policy makers involved with national reimbursement decisions would be familiar with outcomes such as life years gained and QALYs developers of analytics and hospitals deciding on their acquisition may be less familiar with these outcomes but interested in mortality and length of stay. The target population consisted of patients who receive assisted modes of ventilation in a Greek ICU. In current care, IEEVs are not detected in these patients, which means that clinicians do not intervene to stop them. We compared current care with the intervention in which IEEVs are detected with analytics that process data from mechanical ventilators in real-time. Their detection would enable clinicians to provide treatment to reduce duration of the IEEV.

### Decision tree model

**Fig. 1:**
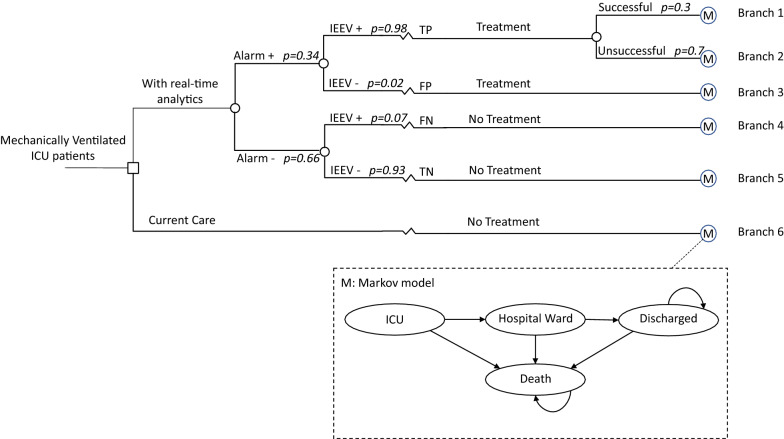
Cost-effectiveness model structure comparing use of real-time analytics to current care. All probabilities were estimated using the sensitivity, specificity and prior probability of having an IEEV reported in this table. *ICU* Intensive Care Unit, *IEEV* Ineffective Effort Event, *FN* False Negative, *TP* true positive, *FP* false positive, *TN* true negative, *M* Markov Model

We developed a decision tree model that compared the health and cost outcomes of current care to the use of analytics for early detection of IEEVs (Fig. 1). In the intervention arm, data from ventilation monitors is analyzed in real-time and an alarm is generated when a patient has an IEEV (branch 1–5). An alarm sounds when patients are labelled as having IEEVs (branch 1, 2 and 3) while no alarm sounds when patients are labelled as not having IEEVs (branch 4 and 5). When the alarm sounds, a clinician will carry out a treatment that may or may not be successful (branch 1 vs branch 2). The other arm in the decision tree represents current care (branch 6). Since IEEVs are currently not identified, no treatment is performed.

Figure 1 also shows a Markov model with four states (‘ICU’, ‘hospital ward’, ‘discharged’ and ‘death’), which was used to estimate the long-term outcomes of IEEV detection and treatment. At the start of this model, all patients start in the ICU. At the end of the first cycle, patients transition to the general ‘hospital ward’ or ‘death’; the cycle length equals the median length of ICU stay. Within the data used to model results, no patients were readmitted to the ICU after ICU discharge. Therefore, we excluded the possibility to transition back to the ICU from the hospital ward.

At the end of the second cycle, all patients in the ‘hospital ward’ transition to either ‘discharged’ or ‘death’; this cycle’s length equals the median length of hospital stay (following ICU discharge). For the remainder of the cycles, patients can remain in the ‘discharged’ state or die; the length of these cycles was one year. Because it was uncertain as to where in the cycle patients transitioned, a half-cycle correction was applied assuming patients transitioned on average in the middle of the cycle. Without the correction, patients would either be assumed to transition at the start or end of a cycle incurring more or less of the costs they should be assigned. The time horizon was lifetime and we adopted a healthcare payer perspective including only direct medical costs. Since Greece does not have a national guideline for performing economic evaluations, health outcomes and costs were discounted at a rate of 3.5%. Key model assumptions can be found in Additional file 1: Table S1 and the model was built in R v.3.3.1.

### Analytics and treatment parameters

Table 1 shows the values and distributions of the input parameters used in the model. Identifying IEEVs and the subsequent treatment can be complex and to estimate its potential several parameters need to be combined. First, ineffective efforts need to be identified from airway pressure and airflow data. In the Greek ICU a prototype monitor was used to identify ineffective efforts. Data from this ‘ineffective effort monitor’ can be used to calculate ineffective effort events. The sensitivity and specificity of the algorithm that identified ineffective efforts were derived from the literature [10]. Real-time analytics would use the data from the prototype monitor to identify clusters of ineffective effort events [5]. The prior probability of IEEVs was 38% [5]. When an IEEV is detected, the clinician can perform one of the following treatments; adjust the ventilator settings, reduce sedation when managing pain and anxiety [7, 8], or change the management of secretions and bronchodilation [8].

**Table 1:** Input parameters for the cost-effectiveness model

Parameter	Base case estimate	Lowest estimate	Highest estimate	Distribution	Source
Discount rate costs (%)	3.5	3	5	–	
Discount rate health benefits (%)	3.5	1	5	–	
Sensitivity prototype monitor (%)	88	79	94	Beta	[10]
Specificity prototype monitor (%)	99	80	100	Beta pert	[10]
Prior probability of IEEVs (%)	38	10	50	Beta pert	[5]
Treatment’s effectiveness (%)	30	0	50	–	[Expert opinion]
ICU survival (%)					
With IEEVs	63	48	77	Beta	Hospital data
Without IEEVs	75	63	84	Beta	Hospital data
Hospital survival (%)					
With IEEVs	41	27	57	Beta	Hospital data
Without IEEVs	67	55	77	Beta	Hospital data
Hazard ratio of death after ICU admission vs no. admission	2.01	1.64	2.46	Normal	[11]
Quality of Life (utilities)					
ICU	0.297	0.24	0.36	Beta	Assumed
Hospital	0.6	0.53	0.67	Beta	[14]
Year 1 post discharge	0.67	0.62	0.71	Beta	[15]
Year 2–10 post discharge	0.70	0.65	0.75	Beta	[15]
Year > 10 post discharge	0.68	0.62	0.74	Beta	[15]
Resource Use					
ICU LOS (days)					
With IEEVs	28	23	34	Gamma	Hospital data
Without IEEVs	22	18	27	Gamma	Hospital data
Time on MV (days)					
With IEEVs	21	17	27	Gamma	Hospital data
Without IEEVs	15	12	17	Gamma	Hospital data
Hospital LOS post-ICU discharge (days)	17.3	14	21	Gamma	[20]
Unit costs (in 2019 Euros)					
Analytics licensing (per bed, per year)	1918	100	20,000	–	[16]
Treatment	100	57	155	Gamma	[Expert opinion]
ICU day	686	392	1060	Gamma	[17]
Hospital day	298	170	460	Gamma	[18]
Reduction in ICU costs when patients no longer receive MV (%)	10	0	35	Beta pert	[Expert opinion]

*IEEVs* ineffective effort events, *ICU* intensive care unit, *LOS* length of stay, *MV* mechanical ventilation, *Hospital data* Patient level data from the intensive care unit of PAGNI in Greece

There is evidence that patients experiencing ineffective effort events have worse outcomes such as increased hospital mortality and prolonged ICU stay [5]. However, assessing the probability that treatments are effective when IEEVs occur can only be done once these real-time analytics are available. Therefore, we assessed the impact on health and cost benefits when varying the probability of effective treatment from 0 to 50%. Because the treatment was performed shortly after an IEEV occurred (3 min) while the median duration of the events was 21 min [5] we assumed that an effective treatment would lead to an outcome similar to those without IEEVs.

### Health parameters

Long term health benefits were quantified in life years gained and QALYs gained. QALYs are estimated by multiplying the life years gained by the quality of life in those years. Therefore, if a patient lives two extra years but in suboptimal health, the QALYs gained will be less than two.

We used patient data on current care from a medical-surgical ICU in Greece (the University hospital of Heraklion (PAGNI)) [5] to estimate life years gained and QALYs. The study was approved by the hospital’s ethics committee and detailed results from the observational study can be found elsewhere [5]. All 110 patients in that study received assisted modes of mechanical ventilation for > 12 h (total of 4,456,537 breaths).

Life years gained were estimated by combining patient level data with results from the literature. The probability of surviving the ICU was considerably higher—although not statistically significant- amongst patients without IEEVs compared to patients with IEEVs (75% vs 63% (*p* = 0.249)). The probability of surviving the hospital was statistically significantly higher for patients without IEEVs compared to patients with IEEVs (67% vs 41% (*p* = 0.025). Life years gained after discharge were estimated using the post-discharge hazard ratio of mortality for ICU patients [11] combined with a baseline hazard of the Greek general population [12, 13].

Unsurprisingly, no research is available on quality of life of patients during ICU stay. Therefore, using a value set from the United Kingdom, quality of life for those in the ICU whilst on mechanical ventilation was assumed to be 0.297. This corresponds with an EQ-5D state of individuals who have extreme problems with mobility and self-care, cannot perform their usual activities but no pain, discomfort or anxiety. QALYs during a hospital stay were estimated using utility estimates derived from the literature [14]. Quality of life after discharge was estimated using the mean age of the patients and the time since ICU discharge [15].

### Resource use and unit costs

To estimate costs, we obtained time on mechanical ventilation and length of stay from the patient level data from PAGNI. For patients with IEEVs, median ICU length of stay was longer than for patients without IEEVs (26 vs 17 days (*p* = 0.017)), as was the median time on mechanical ventilation (16 vs 11 days (*p* = 0.02)). We assumed annual licensing costs for the analytics (€1,918) to estimate the costs of the analytics per ICU day [16]. This estimate was varied extensively in uncertainty analyses. The costs included for treatment when IEEVs occur were assumed to be low since the interventions currently performed to improve interaction between a patient and the mechanical ventilator are easy and cheap to perform (i.e. adjusting sedation, adjustment of ventilator settings). Base case estimates for the costs per ICU day [17] and costs per hospital day [18] were derived from micro-costing studies conducted in Greece. There was a considerable amount of uncertainty in especially the ICU costs per day and these were therefore varied extensively in the univariate uncertainty analyses. These daily ICU costs were decreased by 10% for patients who remained in the ICU but were successfully weaned. All costs were adjusted to 2019 euros.

### Cost-effectiveness analysis

We determined the incremental costs, life years gained, quality adjusted life years and the incremental cost-effectiveness ratio of using analytics to identify IEEVs compared to current care. First, base-case estimates for all outcomes were calculated using the most likely input values based on patient-level data and the literature. We then performed univariate sensitivity analyses in which one input parameter at a time was varied to determine how they affected the cost-effectiveness results. Costs of an ICU day are much higher in countries such as the Netherlands compared to the parameter values used in the base case [19]. Therefore, we assessed the impact of increasing this value to the Dutch estimate (€2153) on the cost-effectiveness results. Finally, we also examined a ‘worst case’ scenario and ‘best case’ scenario using the highest and lowest estimates presented in Table 1. In the ‘worst case’ scenario the analytics and the treatment were expensive, whilst the number of people with IEEVs, the probability of effective treatment, and the sensitivity and specificity of the ineffective effort algorithm were all low. For the ‘best case’ scenario, the analytics and intervention costs were reduced whilst the probability of having IEEVs, the probability of an effective treatment, sensitivity and specificity were all high.

### Probabilistic sensitivity analysis and headroom analysis

In a probabilistic sensitivity analysis (PSA) we varied all parameters simultaneously with the exception of the price and the probability of the treatment’s effectiveness. In the PSA we performed 10,000 simulations during which random parameter values for all input parameters were simultaneously drawn from their underlying distributions. We ran the PSA three times using different levels for the probability that the treatment is effective (10%, 30% and 50%). The results were shown using cost-acceptability curves, which display the probability that using the analytics is cost-effective given various willingness-to-pay thresholds. We also estimated the headroom per patient which is the maximum price that could be charged for the analytics per patient or per bed given a fixed willingness-to-pay and can be estimated as follows;

$$Headroom \, = \, N \, + \, \lambda \, * \, Q$$

where *N* are the savings given a price of zero for the analytics per bed, *λ* is the threshold used and *Q* refers to the incremental QALYs gained [21]. We assumed the device would be sold to a hospital on a per bed basis and that patients needed the device for an average of 17 days. Since no official willingness-to-pay threshold is used in Greece, we adopted three alternative thresholds. The first two were based on opportunity costs proposed by Woods et al. resulting in thresholds of €4,946 and €7758 [22]. Alternatively we also used a threshold of €30,000 which has been adopted in the past in Greek economic evaluations [23, 24].

## Results

### Cost-effectiveness analytics

**Fig. 2:**
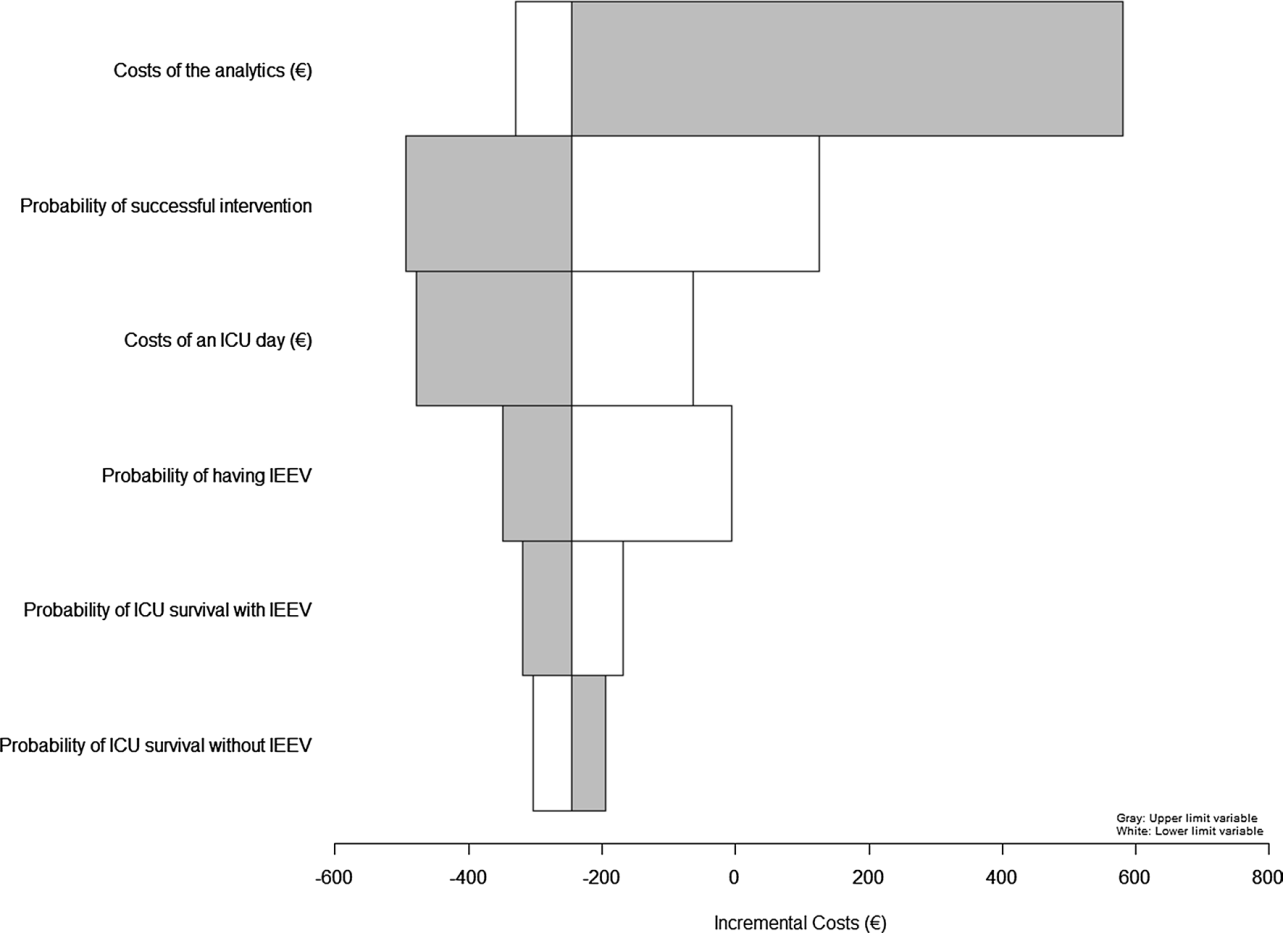
Tornado diagram illustrating the influence of individual parameters on the incremental costs. *ICU* Intensive Care Unit, *IEEV* Ineffective Effort Event

**Fig. 3:**
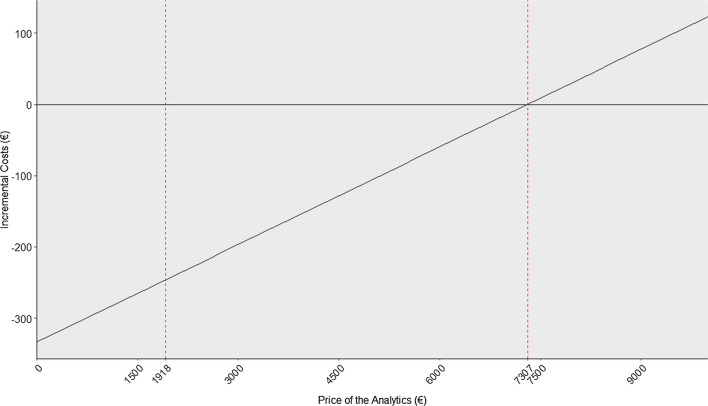
Impact of the price of the analytics on incremental costs

We found that the analytics could reduce hospital mortality (3% absolute reduction), increase QALYs (0.21 per person) and lead to cost-savings (€246 per person) when the probability of the treatment’s effectiveness is 30% (Table 2). Even if the probability that the treatment is effective is small (10%) health improvements and cost-savings were gained. Long-term health outcomes (QALYs and life years) were influenced by hospital survival and the discount rate of health benefits. Incremental costs were greatly influenced by the costs of the analytics, the prevalence of IEEVs, the probability the treatment is effective, and the costs of an ICU day (Fig. 2). Increasing sensitivity and specificity of the monitor that identifies ineffective efforts had a limited effect on costs; but when sensitivity increased so did health gains. In the base-case scenario, when the price of the analytics was €1918, cost-savings were generated (Fig. 3). When the costs of the analytics exceeded €7307 per year, using the analytics was more expensive than current care. Moreover, when costs of an ICU day were high (i.e. €2153), savings increased from €183 to €1155 per patient. In the ‘best case’ scenario, the analytics resulted in greater health benefits (0.50 QALYs), reduced mortality (6% absolute reduction) and higher cost-savings than the base case scenario (€831). However, in the ‘worst case’ scenario, using the analytics offered no health benefits and increased average costs per patient (€895).

**Table 2:** Discounted results from the base case analysis and the worst and best case scenarios

Scenario	Costs €	Length of ICU stay	Hospital Mortality	Life Years	QALYs
Base case					
Current care	19,501	24.28	0.43	6.87	4.72
With analytics	19,255	23.68	0.40	7.18	4.93
Incremental	− 264	− 0.6	− 0.03	0.31	0.21
ICER		Dominant	Dominant	Dominant	Dominant
Worst case^a^					
Current care	18,474	22.6	0.36	7.73	5.31
With analytics	19,369	22.6	0.36	7.73	5.31
Incremental	895	0	0	0	0
ICER		–	–	–	–
Best case^b^					
Current care	19,942	25.00	0.46	6.50	4.46
With analytics	19,111	23.59	0.40	7.22	4.96
Incremental	− 831	− 1.41	− 0.06	0.72	0.50
ICER		Dominant	Dominant	Dominant	Dominant
High ICU day costs^c^					
Current care	53,520	24.28	0.43	6.87	4.72
With analytics	52,366	23.68	0.40	7.18	4.93
Incremental	− 1155	− 0.6	− 0.03	0.31	0.21
ICER		Dominant	Dominant	Dominant	Dominant

*ICER* Incremental Cost Effectiveness Ratio, *QALYs* Quality Adjusted Life Years, *ICU* intensive care unit

^a^High costs of the analytics (€20,000) and treatment intervention(€155), Low probability of IEEVs (0.1), sensitivity(0.79), specificity (0.8) and an unsuccessful treatment intervention (0)

^b^Low costs of the analytics (€100) and the intervention (€57), High probability of IEEVs (0.5), sensitivity(94%), specificity (1) and probability of successful intervention (0.5)

^c^High costs of an ICU day

### Probabilistic sensitivity analysis and headroom analysis

**Fig. 4:**
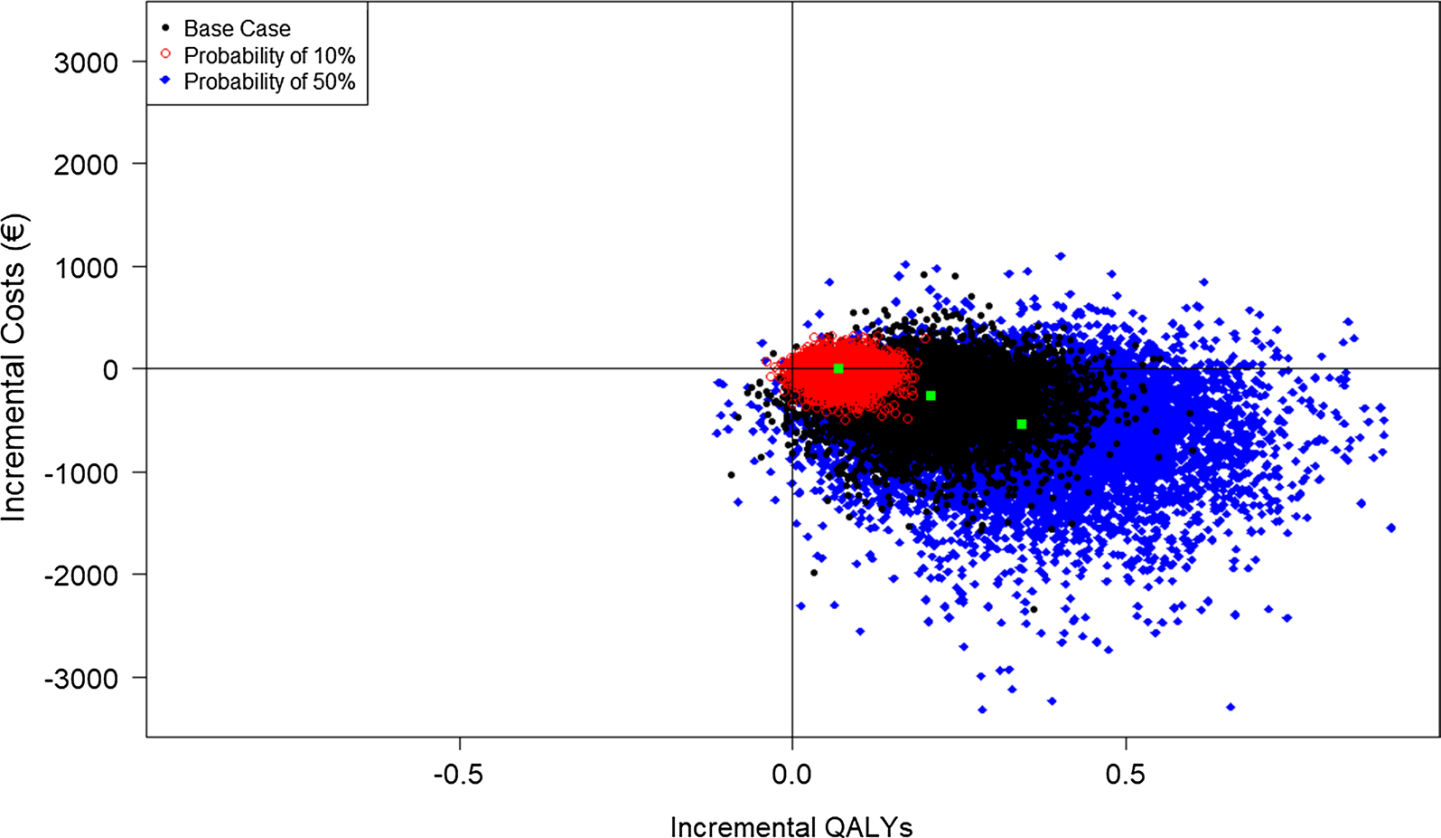
Cost-effectiveness plane for real-time analytics of an ineffective effort event. Results are presented for three probabilities of a successful treatment; 10%, 30% (base case) and 50%

**Fig. 5:**
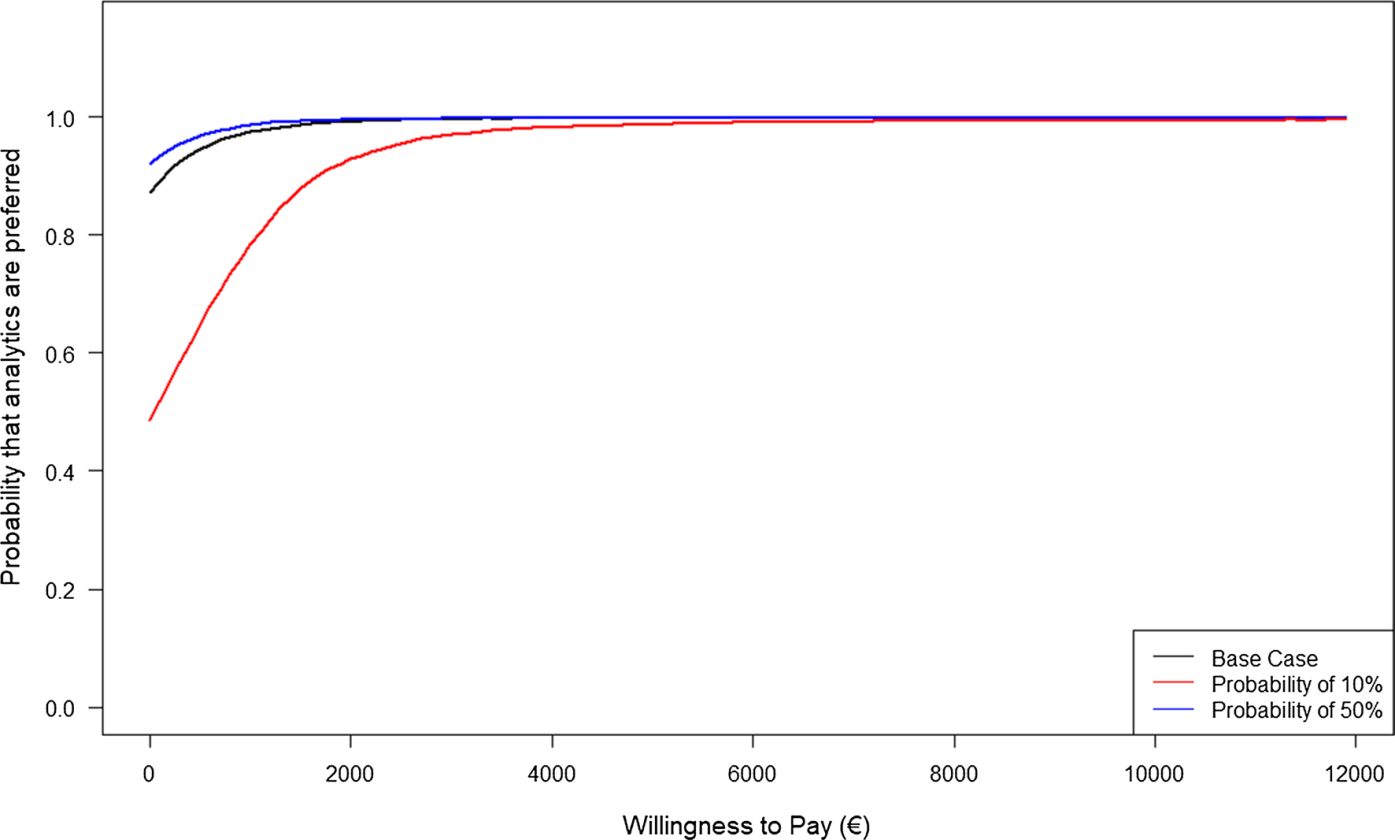
Cost-effectiveness acceptability curves for real-time analytics of an ineffective effort event. Results are presented for three probabilities of a successful treatment; 10%, 30% (base case) and 50%

Figure 4 shows a cost-effectiveness plane that illustrates the degree of uncertainty surrounding the differences in costs and effectiveness between using real-time analytics and current care. Three scatterplots are shown, one for each of the scenarios. This figure shows us that a greater probability that the treatment is effective increases the degree of cost-savings and health gain from using real-time analytics. The cost-effectiveness acceptability curves shown in Fig. 5 present the probability that the analytics are considered cost-effective for a range of willingness-to-pay thresholds. We presented three different acceptability curves each with their own probability of the treatment’s effectiveness. Figure 5 illustrates that for a low willingness-to-pay threshold (€4946), the probability that the analytics for IEEVs are cost-effective exceeds 90% even when the probability that the treatment is effective is 10%. The headroom was €1963 per patient (equivalent to €41,468 per bed), for a willingness-to-pay threshold of €7758. Moreover, for a threshold of €30,000 the headroom per patient was much higher (€6634 per patient equivalent to €140,128 per bed).

## Discussion

We estimated the potential cost-effectiveness of real-time analytics that identify ineffective effort events in mechanically ventilated ICU patients. Even when the probability that the treatment is effective is low, use of real-time analytics could still lead to health benefits for patients (0.21 QALYs per person) and savings (€264 per person) for healthcare payers. Moreover, there is considerable headroom for development since the maximum price that can be charged per bed varies from €28,994 to €140,128 depending on the willingness-to-pay threshold used.

This is the first study to examine the cost-effectiveness of analytics that detect IEEVs. These estimates are important to stimulate further development of analytics that detect IEEVs in real-time since patients with IEEVs have much poorer outcomes compared to those without IEEVs. Previous studies have emphasized that patients with IEEVs have a longer time on mechanical ventilation compared to those without IEEVs and authors have reported that health and economic benefits can be gained by reducing time on mechanical ventilation [25, 26]. Moreover, Marchuk et al. found that those patients with many ineffective efforts in a brief timeframe had reduced oxygen saturation [4]. This further confirms that using analytics that enable timely identification of IEEVs are essential since this allows clinicians to intervene rapidly to improve their oxygen saturation. The underlying assumption that an intervention is successful in at least a small subset of these patients is an important one in the analysis and we cannot be sure that this assumption is valid without further research. However, the results available thus far suggest that it is more likely that an intervention improves outcomes compared to the possibility that the intervention has no or a negative effect. First, we see that patients with IEEVs are severely worse off compared to patients without IEEVs suggesting that there is a lot of room for improvement [4, 5]. Second, IEEVs can be identified after 3 min while their median duration at present is 21 min leaving a large time window in which a clinician can intervene to stop their continuation [5]. This is very important because the potential interventions are relatively easy to perform, are straightforward and are unlikely to lead to any adverse effects. In the unlikely case that there would be absolutely no effect of an intervention whatsoever, we expect purchasers would lose money but patients would not necessarily be worse off. Since the probability of successful treatment influences the health benefits and savings from using real-time analytics, we recommend further development of these analytics for clinical practice and performing a prospective clinical trial to assess their true impact. This study should provide more information about the percentage of patients with IEEVs, and the effectiveness of treating them.

Transferability of our findings to other countries and hospitals could be influenced by the cost estimates used in our analyses. Especially ICU costs had a large influence on the results and we therefore varied these costs by 25% in the univariate sensitivity analysis. Moreover, we also performed a scenario analysis using the ICU costs of the Netherlands as an example for other western countries. The benefits for hospitals also depend on the reimbursement system in place. Diagnostic related groups in which hospitals receive a fixed payment for patients with a specific diagnosis can stimulate hospitals to reduce length of stay which could in turn lead to financial savings for hospitals. However, if services are reimbursed on a fee-for-service basis in which the hospital is reimbursed for each additional day in the hospital, there could be perverse incentives to increase length of stay. Either way, the aim of healthcare providers should be to maximize the health outcomes of their patients which makes use of analytics to detect IEEVs desirable. We excluded the possibility that alarms generated by the analytics might sometimes be ignored because of alert fatigue which could lead to lower benefits than estimated here. We also excluded the possibility that patients are readmitted to the ICU and excluded any side effects of treatments to stop an ineffective effort event. Even though no patients were readmitted in the observational study and experts thought that side effects did not necessarily occur, both should be verified in a clinical trial.

Our results are not generalizable to all ICU patients receiving mechanical ventilation, since we only considered patients who were expected to remain on proportional assisted mechanical ventilation for a longer period of time (> 24 h). Furthermore, a small subset of patients can have IEEVs a couple of days after initiation of ventilation support. Our assumption that all treatments are performed on the first day could therefore have led to an overestimation of the benefits of using the analytics. Even though few patients had IEEVs after the first day, additional research on the estimated number and timing of IEEVs could improve the estimate of the benefits. A final limitation is that we did not include any benefits of reducing any delays in ICU admission of other patients. Since there is a shortage of ICU beds in Greece, reducing length of stay for patients with IEEVs could reduce health losses incurred by other patients because of delays in admitting them to the ICU. Therefore, the true benefits could be higher than presented here.

Although clinical experts have emphasized the relevance of developing analytics to detect IEEVs [5, 27] their adoption is uncertain and compromised by constrained budgets and competing investments. Our results provide developers with estimates of the potential benefits of these analytics, which they can show to healthcare payers. There is a considerable market that could benefit from analytics that identify IEEVs since the number of critical care beds in Europe has been previously estimated at 75,585 [28]. Sixty percent of all ICU patients receive mechanical ventilation, of which 30% will receive prolonged ventilation [29, 30]. Therefore, the analytics would be relevant for 18% of ICU patients. In Greece, there is a shortage of ICU beds and because of this all ICU beds are constantly occupied. If this is also the case in other European countries, the analytics would be relevant for 18% of these 75,585 beds in Europe alone. Based on our results, the analytics should first be assessed in countries where ICU costs are high, such as the United States or The Netherlands, where the potential financial benefits of the analytics would be considerably higher.

## Conclusion

Real-time analytics to identify ineffective effort events have the potential to improve patient outcomes and generate financial savings for healthcare payers even when the probability of an effective treatment is low. There is considerable headroom for development and this should therefore be encouraged. Exploitation in countries where the costs of an ICU day are high could yield a higher return on investment. One important next step is to obtain additional clinical evidence of using these analytics in settings where there is a high frequency of IEEVs.

## Supplementary Information

**Additional file 1: Table S1.** Overview of key assumptions underlying the model.

## Data Availability

The datasets used and/or analyzed during the current study are available from the second author (KV) on reasonable request.

## Abbreviations

ICU: Intensive care unit
IEEVs: Ineffective effort events
QALY: Quality adjusted life year
ICER: Incremental Cost Effectiveness Ratio
PSA: Probabilistic sensitivity analysis
