# Supplementary material for: The potential of real-time analytics to improve care for mechanically ventilated patients in the intensive care unit: an early economic evaluation
Source: Cost Eff Resour Alloc. 2020 Dec 11;18:57. doi: 10.1186/s12962-020-00254-4 (PMC7729701; doi:10.1186/s12962-020-00254-4)
Supplement: Supplementary file 1 — Additional file 1: Table S1. Overview of key assumptions underlying the model. [file 12962_2020_254_MOESM1_ESM.docx]

The potential of real-time analytics to improve care for mechanically ventilated patients in the intensive care unit: an early economic evaluation

**Table S1. Overview of key assumptions underlying the model**

| Assumption | Potential Impact |
| --- | --- |
| We assumed patients do not transition back to the ICU during their initial hospital visit. | If the number of patients transitioning back to the ICU during the initial visit is high we may have underestimated the costs in care with the analytics. With the analytics more patients survive their initial ICU visit and thus more of these patients are eligible for a readmission and thus higher costs. However, this would only be the case if the intervention performed when IEEVs occur has no impact the risk of readmission |
| We assumed a base case effectiveness estimate of 0.3 | This estimate was varied very extensively in the uncertainty analysis. Therefore, the potential impact of this assumption is clearly demonstrated throughout the paper. Of course it is crucial to perform a clinical trial once real-time analytics have been developed. |
| We assumed patients in the ICU have a utility estimate of 0.297 which was based on no pain, discomfort or anxiety | It is possible that when sedation is adjusted when an IEEV occurs, a clinical expert has difficulty finding the balance between a patients ability to trigger the ventilator (so no excessive sedation) while avoiding pain/anxiety. However, if pain or anxiety occurs (thus the balance has not been found) it is unlikely that patients will experience this for very long because the clinician will continue adjusting medication until this balance is found. |
| We assumed costs of the analytics to be €1918 | This estimate was varied very extensively in the uncertainty analysis. Therefore, the potential impact of this assumption is clearly demonstrated throughout the paper. |
| We assumed costs of the intervention to be €100 | At present all interventions that are applied to improve the interaction between a patient and a mechanical ventilator are quite simple and relatively low in costs. We therefore consider this assumption reasonable. Would these costs be higher in reality we may have slightly overestimated the savings. |
| We assumed patients used the analytics for 17 days on average when estimating the headroom per bed and that all beds were constantly occupied (thus a shortage of ICU beds). | This estimate was based on the results from the observational study performed in PAGNI and the shortage of ICU beds in Greece. It is possible that in other European countries the headroom would be slightly lower because there is no shortage of ICU beds. Furthermore, we expect that ICU stay could be shorter in other countries for instance because of differences in quality of care and patient characteristics. |
